# Mental illness and suicidality among Roma and traveller communities in the UK, Ireland, and other countries: a systematic review

**DOI:** 10.1186/s12888-025-06752-0

**Published:** 2025-04-04

**Authors:** Arav Dagli, Roger T. Webb

**Affiliations:** 1 Manchester Medical School, Faculty of Biology, Medicine, and Health, The University of Manchester, Manchester, M13 9PL UK; 2 Division of Psychology and Mental Health, The University of Manchester, Manchester Academic Health Science Centre (MAHSC), Manchester, UK

**Keywords:** Gypsies, Romas, Romanis, Irish travellers, Mental illness, Mental health, Suicidality, Depression, Anxiety

## Abstract

**Background:**

Romas and Irish Travellers are two distinct, traditionally nomadic ethnicities of people who experience lower socioeconomic position and social exclusion. This occurs within the context of long-term attempts to maintain their traditional culture. They are known to have significantly worse health outcomes than the general population. This systematic review identified and appraised the existing literature on the mental health status of Romas and Travellers, as well as suicidality in these communities. It aimed to determine how their mental health status compares to that of the general population and propose mechanisms for any differences observed.

**Methods:**

All databases on OVID were searched using three search strings for relevant articles, which were then manually screened to ensure that they were relevant. All studies included were assessed for quality standards.

**Results:**

The evidence shows that Romas/Travellers have a far higher prevalence of mental health problems than the general population. The most recent evidence suggests Romas/Travellers have a higher rate of suicide compared to any other ethnicity in the UK. A range of factors were associated with this disparity, including socioeconomic deprivation (issues with housing, education/employment, and discrimination), poor physical health, and barriers to accessing healthcare. Women faced worse mental health outcomes, possibly due to enforced gender roles, early marriage, and domestic violence.

**Conclusions:**

Romas/Travellers face higher rates of mental illness and suicidality, which is largely multifactorial in nature. Significant stigma around mental health and suicide still exists in these communities, making it difficult for affected individuals to seek help. Community-based, targeted interventions are urgently needed to alleviate the harmful impacts of poor mental health and suicide on these communities.

**Supplementary Information:**

The online version contains supplementary material available at 10.1186/s12888-025-06752-0.

## Background

### Identity and culture

Roma people, those of Romani ethnicity, are traditionally nomadic people who originated in modern-day India. Due to migrations, their population is now largely centered in Europe, where they form the largest ethnic minority. It is thought that they arrived in Europe around the beginning of the fourteenth century [1]. The term “gypsy”, often used by non-Roma people to refer to them, can in many instances be offensive and stigmatising; for this reason, the term “Roma” is generally used in this study, as this is the term that was chosen by the Roma people during the World Roma Congress in 1971 [2]. Large numbers of Roma people live in Romania, Bulgaria, Slovenia, Slovakia, Macedonia, Czech Republic, and Hungary. There are many Roma in the UK as well, largely in England and Wales [1].

Romas form a separate ethnicity from Irish Travellers, who are another nomadic ethnicity indigenous to Ireland [3]. They do not share ancestry with the Roma and are instead descended from the same ancestors as the settled Irish population [3]. Contrary to popular belief, Irish Travellers did not emerge as a response to the Great Famine in the 1840s; rather, they split from the settled Irish population around 360 years ago [4]. Irish Travellers are found in large numbers only in Ireland and the UK. The majority of Irish Travellers are Roman Catholic by faith [3].

Due to their nomadic nature and the absence of census returns from many members of these communities, it is hard to determine the size of their population. The UK Census draws a differentiation between British Gypsies/Irish Travellers and Roma people, by defining the Roma as more recent immigrants from Central/Eastern Europe (whereas Gypsies are those with stronger ancestral roots in England). However, on most other government documents and projects, they are grouped together under the term Gypsies, Roma, and Travellers (GRT). The 2021 Census returned a count of 71 440 Gypsy/Irish Travellers and 103 020 Roma in England and Wales, but estimates from 2011 suggest that the true population could be up to 500 000 [5, 6]. In Ireland, the 2022 census found that there are 32 949 Travellers, but the government’s National Social Inclusion Office states that this count is not complete [7]. Many Romas and Travellers still maintain an itinerant lifestyle, especially around the times of seasonal weather changes [1]. However, they are becoming more settled, both due to changes in modern society and institutional pressures that have pushed them toward giving up their nomadic lifestyle [1]. In the UK, around 75% of GRTs live in brick-and-mortar houses, with the remaining 25% living in mobile structures such as caravans. However, many who have settled continue to remain nomadic for part of the year before returning to their fixed abode [8].

Despite a distinct ancestral heritage, Roma and Traveller cultures are similar in many ways. A strong emphasis is placed on family and on the immediate collective community, which often tends to be made up of an extended family or small groups of families [9]. This collective is the unit within which resources are shared and labour is divided. They tend to marry younger, and have larger families, than the general population [9]. Elder members of the collective are seen as authoritative figures, and their commands or decisions are usually accepted without question [10]. Beyond this small community, many Roma/Travellers associate themselves with a clan or nation, which may identify itself based on criteria such as dress, dialect of language, trade, or location [9]. Cleanliness, both physical and figurative, is very important in the Roma/Traveller culture; certain concepts can be seen as unclean and therefore unfit to be discussed with others, even family. These include the lower half of one’s body, menstruation, and sex [9]. Both communities also practice traditional folk medicine, which can make it difficult to engage them in modern healthcare programmes [3, 11].

Gender roles tend to be rigid in these communities, with a patriarchal structure prevailing [12]. After marriage, men are typically the sole earners, whereas women are primarily responsible for the home and the care of children/elders [12]. Strict rules of etiquette are expected of women, and deviation from these rules can bring dishonour to them and their family [12]. Based on qualitative research, this gender divide is largely accepted by both men and women as not only normal, but an integral part of the culture [12]. This makes it harder for the harms that emanate from them to be rectified [13].

Romas and Travellers also have socioeconomic similarities. They are both disproportionately low-income, with low educational attainment in adolescence and high unemployment rates in adulthood [14, 15]. Difficulties surrounding access to key infrastructure, and a lack of support from governments and settled society, hinder the socioeconomic development of this community.

### Prejudice, and systemic hardships

Due to a UK-wide shortage of available caravan sites, which the government has tried and failed to address, unauthorised land is increasingly being used to house GRT communities [8]. These camps cause tension between GRTs and settled society and contributes to the overall negative view of GRT communities that is held by a large portion of mainstream UK society [8]. Racism towards Romas is commonplace and is largely fueled by prejudiced and negative coverage in mainstream media [16]. Stereotypes that are commonly associated with Romas populations include thievery, dirtiness, poverty, and violence. They are also often seen as lazy, using services and land that is not theirs without working or paying taxes [16]. It is commonplace for GRT individuals to feel as though they must hide their ethnicity to avoid racism and discrimination, especially when applying for jobs [16]. In the UK, GRTs regularly report being harassed not only by the local populace, but also by authorities, leading to a very strong feeling of distrust for the settled community and their institutions [17]. In 2006, 67% of local authorities said they’d had to mitigate tension between GRT and the general public, with 94% of these cases being due to unauthorised encampments [17].

A 2018 survey found that 44% of the British public express negative views about GRTs [8]. It has been noted by researchers is that while racism to other communities is widely viewed as being socially unacceptable and is therefore hidden, racism towards GRT communities is still seen as justified and is often overt [17]. Although racist attacks directed at GRTs are commonplace, they are not widely reported to the police. The main reasons for the lack of reporting are that the incidents are too common, and the belief that police will not do anything to resolve them [18]. Irish Travellers face similar experiences in Ireland, with widespread reports of Travellers needing to change their accents and attire and conceal their identity to enable them to access basic services and facilities [19].

### Health status

Studies have found that GRT populations have worse physical health than the general population [20–23]. GRT populations have lower levels of exercise and poorer diets, with particularly insufficient levels of fruit and vegetable intake [24]. GRT men are at higher risk of premature death due to cardiovascular disease. In the EU, Roma individuals have a life expectancy that is between 5–20 years shorter than the general population; in the UK, life expectancy for GRTs is 11 years shorter [25]. In Ireland, male Travellers have a life expectancy 15 years shorter than the general population, while female Travellers have a life expectancy 11.5 years shorter [26]. People from Roma and Traveller communities are likelier to develop long-term conditions and infectious diseases [17, 26, 27]. Despite this, GRT communities access healthcare far less than the general population, for a variety of reasons that will be explored later in this review [28].

The information provided above paints a picture of a community that is largely isolated from the rest of society, misunderstood by the public, and difficult to engage in healthcare due to a myriad of factors. In the pursuit of upholding their traditional culture, lifestyle, and values, they face socioeconomic deprivation and formidable institutional barriers. These hardships and challenges yield health outcomes that are significantly worse than the general population. For these reasons, this review aims to systematically review the existing literature on mental health and suicidality in GRT populations, both in the UK and Europe. It aims to discuss the prevalence of mental health issues and suicidality and uncover GRT attitudes towards mental health. It also aims to formulate ideas as to why people in these populations are more likely to experience higher rates of mental illness. A more comprehensive understanding of these topics would be useful in planning interventions to attempt to narrow the disparity in mental health that is evident among GRT communities.

## Methods

### Search strategy

Ovid® was used to search for papers, with all databases on the platform being selected. This was intentionally done due to the nature of the subject matter, which has many social and psychological components as well as medical ones. By not restricting the search to solely a medical database, papers were found not just from the medical journals, but also from social sciences and applied health sciences journals.

Three searches were done, which differed only in the line of the search that was included to identify articles specific to Roma/Traveller people. One search was done using the Medical Subject Heading (MeSH) term “Roma,” while a second was done by manually searching for all the relevant terms for Roma people, with spelling variations included as well. The third search was done to search for papers relating to Irish Travellers. In the MeSH search, the “Roma” term was exploded, allowing it to search for papers that discuss Roma or any of the related terms that are listed under this subject heading. In the search without a MeSH, the terms that were searched for were restricted to the title/abstract, with the intention of only drawing papers where mental health and Roma people were a key focus.

The search string that did not use a MeSH term for Roma people read as follows:

1. ("mental health" or "mental illness" or "mental disorder" or "suicid$" or “self-harm*” or “self-injur*” or “self-poison*” or “self-inflict*” or "psychosis" or "depress$" or "anxiety" or "bipolar" or "schizo$").ti,ab.
2. ("gypsy" or " gypsies" or "gipsy" or "gipsies" or "roma" or "romani" or "romany" or "rromany").ti,ab. not IBS not "irritable bowel" not graft* not "roma criteria”
3. 1 AND 2

The terms that follow the “not” operator were used to rule out specific meanings of the word “Roma” that did not relate to Roma people. An example of this is the ROMA trial discussing coronary artery bypass grafting [29]. A similar difficulty that was encountered but was unable to be dealt with at this stage, was the city of Rome in Italy; this was often referred to as Roma in articles, especially those by Italian authors or in Italian journals. As there was no way to use the “not” operator to exclude these without excluding any Italian articles (where studies on Roma people could have been conducted), these were kept in the search and removed manually.

The search string used to find papers relating to Travellers read as follows:

1. ("mental health" or "mental illness" or "mental disorder" or "suicid$" or “self-harm*” or “self-injur*” or “self-poison*” or “self-inflict*” or "psychosis" or "depress$" or "anxiety" or "bipolar" or "schizo$").ti,ab.
2. “Irish Travellers”ti,ab.
3. 1 AND 2

The search string that used a MeSH term was utilised as follows:

1. exp *Roma/
2. (mental health or mental illness or mental disorder or mental disease or depressi* or anxiety or bipolar or psychosis or schizo* or suicid*)
3. 1 AND 2

### Inclusion/exclusion criteria

The set of inclusion/exclusion criteria was discussed and finalised by both authors jointly. Publication time period was not an assessed criteria due to the lack of high-quality research on the topic of this paper. However, older studies were interpreted with this in mind. Once these criteria were finalised, AD was responsible for applying them to studies initially identified by the search. When it was unclear whether a paper met the criteria or the scope of this systematic review, a joint discussion took place between the authors (AD and RTW) make a final decision. This resulted in a consensus decision after weighing up the attributes and limitations of the study in question.

### Selection of papers

**Fig. 1:**
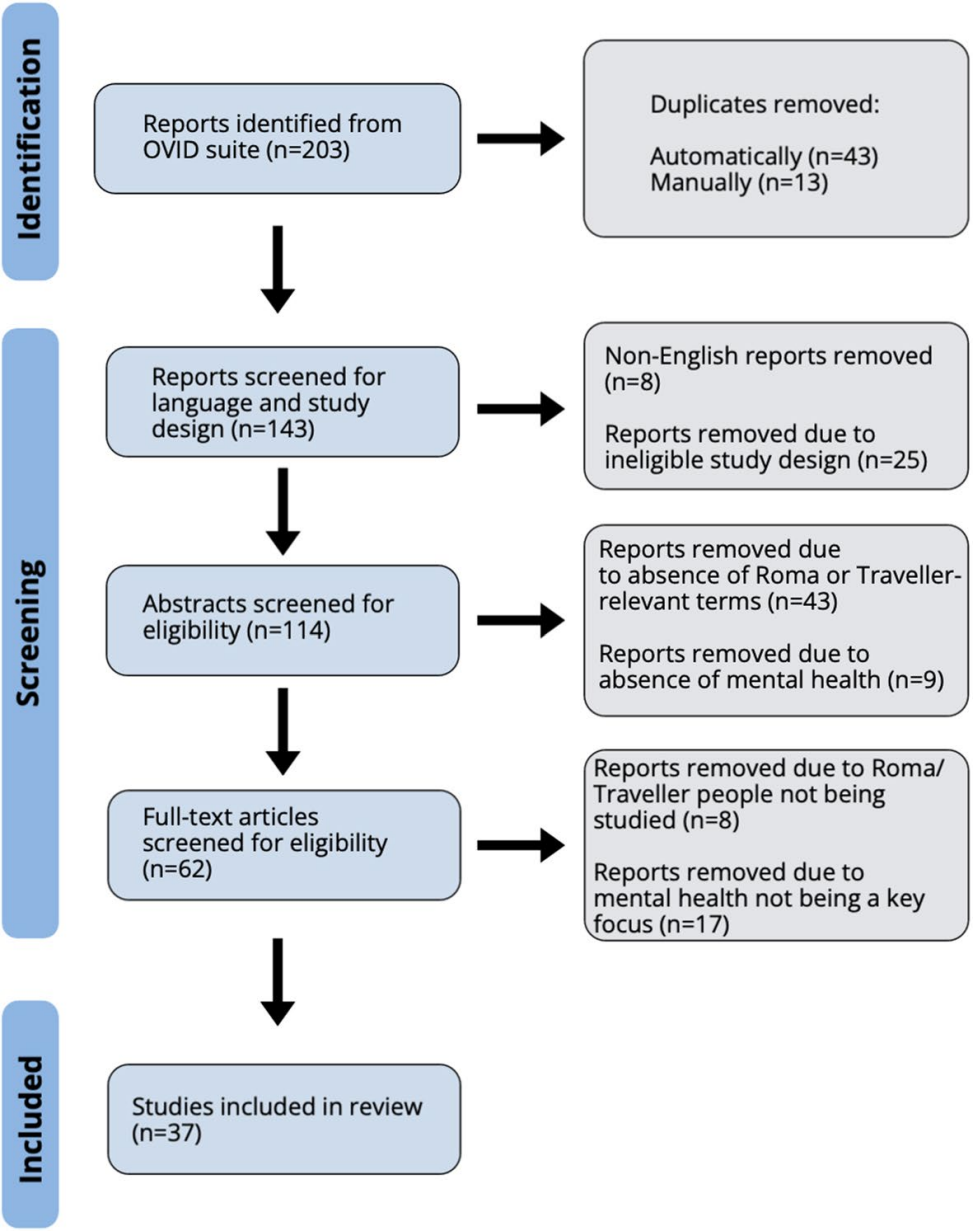
A PRISMA flowchart showing the study selection process

Combining both the MeSH search (n = 15), the Roma non-MeSH search (n = 125), and the Irish Traveller search (n = 62) a total of 203 studies were drawn from the databases. After importing these into a citation manager, 43 duplicates were automatically removed and a further 13 were removed manually. 147 reports were then screened for eligibility. 8 reports were removed for being in a foreign language, and 25 were removed for not having eligible study design (see Table 1). This left 114 abstracts to be screened. 43 of these were removed for not mentioning Travellers or using one of the terms for Roma people, or for using one of these terms to refer to something other than the Roma people. 9 were removed for having no mention of mental health. 48 full text articles were screened for final eligibility. Of these, 8 were excluded for not studying Roma or Irish Traveller people directly. Some of these were studying people’s perceptions of Roma/Traveller people, whereas others were studying various ethnicities and did not have enough of a focus on Roma/Traveller people. Lastly, 17 articles were excluded for not having mental health as a key focus of the article. This left 37 articles to be included in the systematic review. A detailed PRISMA flowchart of the selection process can be seen below. Figure 1.

**Table 1:** A summary of the inclusion and exclusion criteria used to shortlist articles, shown in a step-by-step manner

Selection Stage	Criteria Assessed	Included	Excluded
**Database search**	Language	English	Non-English languages
	Study Design	Any form of primary research published in an academic journal	Case studies, reviews
**Abstract Screening**	Roma	One of the terms for Roma people is present, and used to refer to Roma people	None of the terms for Roma people are present, or they are used to refer to something other than the Roma people
	Mental Health	There is mention of mental health or suicidality in the abstract	There is no mention of mental health or suicidality in the abstract
**Eligibility Assessment**	Roma	Roma people are being studied	Roma people are not being studied, or people’s perceptions of Roma people are being studied
	Mental Health	Mental health is a key focus of the paper	Mental health is not a key focus of the paper

### Pre-registration of protocol

A pre-registration of the systematic review protocol was not completed, as this manuscript was initially prepared for a medical school project. The two authors decided to submit the manuscript for publication after it was already written. As the manuscript was ready for submission at this point, the decision was made to not pre-register the protocol.

## Results

### Quality appraisals

Quality appraisal checklists from the Joanna Briggs Institute (JBI) were used to assess each study’s overall quality according to the study type [30]. These checklists included tick boxes for different criteria of study design, such as sampling frame, sample size/response rates, and statistical analysis [30]. All the studies included fell into five groups of study type: prevalence studies, analytical cross-sectional studies, cohort studies, case–control studies, and qualitative research. The full versions of these checklists, including the lists of numbered criteria that were applied with each specific checklist type, can be found in Additional File 2. Table 2.

**Table 2:**
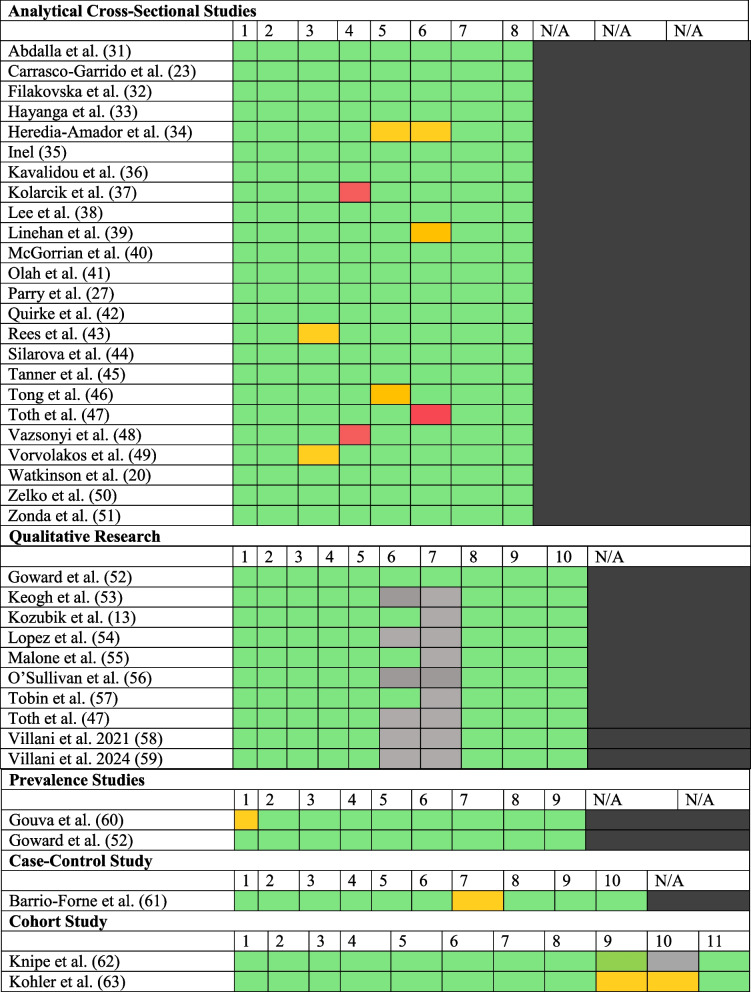
The results of the JBI checklists [13, 20, 23, 27, 31–63]^a^

^a^For each criterion, denoted by the number that corresponds to it on that design’s checklist, green indicates an answer of “Yes”, red “No”, amber “Unclear”, and grey “Not Applicable”.

### Results from studies

An additional table shows the main information found in each study, along with the country the studies were done, sample sizes, study designs, and method used to quantify mental health [see Additional File 1].

### Narrative synthesis

The studies included in the reviews largely showed that Romas and Travellers have worse mental health outcomes than people in the general population [20, 23, 27, 31, 33, 34, 36, 40, 44, 49, 50, 52, 60, 64]. A study from the UK showed that of all ethnicities, Romas had the highest risk of having a long-term mental disorder [33]. Another UK study similarly showed that Romas had the worst levels of anxious/depressive symptoms of any ethnicity [20]. However, mental illness appears to be underdiagnosed in this population [20]. A study found that although those who attempted suicide multiple times had severe depressive signs, less Romas had a diagnosis of depression than Hungarians [41]. A significantly larger portion of the Traveller population was found to experience frequent mental distress compared to the non-Traveller population [40]. Qualitative interviews with Travellers showed that they believe socio-economic factors like income, education, and housing, as well as prejudice, play an important role in the development of mental ill health [42, 58].

Several studies found that Romas also have worse physical health and health-related quality of life, both of which are associated with their mental health status. A UK-based study showed that Romas had the worst health-related quality of life of any ethnicity; this was equivalent to a 20-year increase in the age of the entire population [20, 50, 52]. Roma patients with mental health issues were found to have worse quality of life than those with musculoskeletal and cardiac health issues [50]. Romas are also the ethnicity with the highest prevalence of multiple long-term health conditions in the UK [33]. They also scored lower on self-rated life satisfaction and well-being [64].

Roma women are particularly affected by negative mental health outcomes. Studies have found that they have worse outcomes than Roma males [50, 60]. When compared to non-Roma women, they are disproportionately anxious during pregnancy [61], and have a higher level of perceived stress when raising their children [32]. Roma women who experience domestic violence have high rates of psychological problems [13]. In the population of Roma women who marry before the age of 18, the duration of the marriage is associated with more mental health issues [35].

Evidence for mental health outcomes of adolescents was mixed; some studies suggested that Roma children have worse mental health outcomes than the general population [32, 38] and some suggested that there was no difference [37, 48]. A study from Romania & Bulgaria found that Roma adolescents had higher rates of externalising issues [38]. Another found no link between Roma ethnicity and externalizing issues [48]. High stress on Roma mothers was linked with harsher disciplinary methods, which are linked with worse mental health outcomes for children [32]. Qualitative interviews with Travellers suggested that many believe that adverse childhood experiences, particularly discrimination and bullying in school, is where mental health issues in the Traveller community start [58].

A study from 1990 found that Roma people attempt suicide more often, but die by suicide less often, when compared to the general population, and they are likelier to have multiple attempts [51]. Travellers follow a similar trend, with multiple studies finding that they are far likelier than the general Irish population to self-harm or attempt suicide [31, 36, 45]. The intent behind the attempt also differs, with Romas being less likely to attempt with the intent to die. However, a study from 2024 found that Gypsies and Irish Travellers in England and Wales have the highest suicide rate of any ethnicity, casting the findings from 1990 into doubt [62]. Further, higher quality epidemiological research is needed to clarify the direction of this association. More often, Romas mean to escape a situation or appeal to others, and they are likelier to attempt suicide impulsively [47, 51, 62]. A Bulgaria-based study showed a religious divide, with Christian Romas being the most likely population to die by suicide and Muslim Romas having relatively lower suicide rates. This was despite the two populations being comparable in other domains such as socioeconomic position and physical health status [63]. A joint science-arts exhibit on the effect of suicide on Traveller communities in Ireland was found to be effective at engaging the community in a discussion about this otherwise stigmatised and undiscussed topic [55].

## Discussion

### Summary

In summary, the evidence shows that Romas and Irish Travellers have significantly worse mental health outcomes than the general population; this is a theme that was common to all countries studied. This trend is particularly marked in women, but more unclear in adolescents and children. There is a link between Romas’ physical health and health-related quality of life, which are also both worse than the general population, and their mental health. Romas and Travellers attempt suicide more often. Romas are likelier to attempt suicide multiple times, but evidence varies between studies on whether they are more or less likely to die by suicide. Research conducted more recently in the UK has shown that Romas have a higher suicide rate than the general population. Romas are also found to be likelier to impulsively attempt rather than deliberately planning an attempt. Mental health is shown to be affected for both communities by socioeconomic factors as well as discrimination and prejudice from the general population. Before interpreting these findings, it is important to note that the quality of evidence is limited, with most studies being cross-sectional or prevalence studies and with very few cohort studies. This dearth of high quality evidence may reflect the specific challenges in conducting research on these groups as well as lack of available funding.

### Interpretation

#### Socioeconomic factors: housing, discrimination, and income

A major factor associated with mental health in many of the studies was housing. The physical environment and housing situation of Roma and Traveller communities makes up a large part of their connectedness with their culture. Therefore, housing situations that are seen as unideal can have a large impact. It has been acknowledged by the UK government that there is a shortage of sites where Roma/Traveller communities can stop with their mobile residences. The 1994 Criminal Justice and Public Order Act took away the consequences that local authorities would be subject to if they did not provide public sector sites for Roma/Traveller populations. As a result, these authorities have not been meeting the need for caravan pitches where Roma/Traveller populations can legally and safely stop [65]. As of 2021, there were only 59 permanent pitches and 42 temporary pitches available in all of England, with 1696 families on the waitlist for somewhere to live [66].

As a result of this situation, Roma/Traveller families in the UK are increasingly being forced to stop their caravans on unauthorised land [8]. Local authorities often evict these unauthorised roadside camps within days, forcing them to move on in search of somewhere else, where there is risk of the same incidents occurring again [67]. Similar incidents occur across Europe. In May 2024, the government of Italy was found to have violated the European Social Charter in its discriminatory housing policies towards Roma communities [68]. In the Czech Republic, a half of Romas have reported feeling threatened by evictions. Even during the COVID-19 pandemic, where moratoria against evictions were in effect, evictions of Romas continued [69]. Nomadic Romas are made to move between various unstable locations, unable to spend any prolonged period in one spot. These forced displacements are unsustainable and can cause a deep sense of vulnerability. The inability to live life according to the cultural ideals that have been a formative part of one’s identity can be very damaging to one’s mental health [70]. Spurred by the feeling that cultural change is being forced on them, and that there is a deliberate, powerful attempt to destroy their way of life, Romas/Travellers in the UK and Ireland are becoming increasingly anxious and depressed [53, 67].

On the other hand, the legislative and systemic difficulties associated with maintaining their traditional lifestyle makes many Romas/Travellers feel forced to give up travelling altogether. They are pressured into attempting to assimilate into settled society, which also leads to worse health outcomes. Parry et al. found that living in a house or council site was associated with higher prevalence of long-term illness and higher severity of anxiety. Furthermore, Romas/Travellers who rarely travelled were found to have the worst health status and health-related quality of life [27]. In Ireland, policy changes over the last few decades are seen to have contributed to a growing feeling that the nomadic way of life is becoming criminalised, and this is eroding Travellers’ sense of self-worth [58].

Another issue altogether is the quality of the housing that Romas do receive, both those that are on the move and those who attempt to settle. Encampments are often located in areas with poor sanitation and little access to drinkable water [8]. In the European Union, local authorities have been known to use forced evictions and house demolitions as tools to rid municipalities of their Roma inhabitants [69]. What results is the ghettoization and isolation (both physical and social) of Roma communities. Restricted to the outskirts of their municipalities, they form segregated settlements in areas with substandard conditions and a lack of basic essential infrastructure [69]. In Ireland, there are similar phenomena; Travellers believe issues with housing, including forced settling and unsuitable conditions, are a key contributing factor towards developing poor mental health [53, 58].

Being able to choose where to stay, and when to leave, gives people a sense of control over their own lives. Forced to constantly move or to settle, Romas feel this sense of control is collapsing; this is especially true when attempts to assimilate are met with more hostility [67]. It erodes their ontological security, or the aspect of their wellbeing stemming from a sense of stability in, and control over, one’s own life and environment [71]. This fundamental lack of security and peace in their physical environment may be a large driving factor behind the poor mental health outcomes that this community faces. This idea is supported by findings from Olah et al., where ethnicity was not found to have any effect on mental health once socioeconomic factors were accounted for. The single largest factor that was correlated with a poor mental health was living in a segregated settlement [64]. Vazsonyi et al. also showed that concerns about neighbourhood safety is associated with anxiety [48].

Discrimination is not just limited to housing policies; Romas and Travellers are regularly targeted by hate crimes, including hate speech and racist attacks [18, 53]. Travellers and Romas report being excluded from services, both essential and recreational, because of their ethnicity. There are widespread reports of Romas/Travellers feeling that they must hide their ethnicity to participate in society, keep employment, and avoid discriminatory attacks [53, 58, 72]. Discrimination was seen as the strongest predictive factor of poor mental health in Traveller populations [73], This is widely acknowledged by Travellers themselves, who believe the regular prejudice and hate they face has had a negative impact on their mental health [53]. Additionally, Quirke et al. found that a large portion of Travellers feel they and the general population had equal access to mental healthcare. However, those who did not feel this way were significantly likelier to have experienced discrimination from the general population [42]. This shows that not only does discrimination directly impact mental health, but it can also create a lack of trust in the healthcare options that settled society has in place for Travellers to get help. To add to the issue, the segregated settlements that they are forced to live in provide them with virtually no opportunity to integrate into mainstream society, reinforcing their status as outsiders and worsening the discrimination [69].

This segregation from settled infrastructure also creates a cycle of poverty, subjecting entire communities to lives of little to no education and low income. In the UK, Roma and Traveller pupils have the lowest educational attainment of all ethnicities, and are the least likely to stay in school after GCSEs [14]. The 2016 Irish Census shows a similar trend for Travellers in Ireland; 28% of Travellers over the age of 25 had left school before the age of 13, compared to just 1% of the general population [74]. This translates into a lack of employment. A European survey in 2021 found that 57% of Roma are unemployed, compared to the EU average of 28% [15]. Their economic status reflects the same; the survey also showed that 80% of Roma are at risk of poverty compared to the EU average of 17% [15]. Lower socioeconomic position is associated with negative mental health effects, and persistent low income is strongly related to higher rates of mental health problems [75]. In qualitative interviews with Travellers, a common theme that emerged which provides more evidence for this point. Lack of education, unemployment, and the resulting financial instability significantly contributes to the mental distress that Travellers experience, especially men [53, 58]. A lack of education, employment, and income also allows negative stereotypes about Roma to be reinforced.

Roma communities’ housing difficulties, lower sociodemographic position, and experiences of discrimination all exacerbate each other, creating a campaign of social exclusion. These socioeconomic factors alienate an already vulnerable community, and this has negative effects on mental health. Social exclusion and alienation can be associated with higher severity and frequency of depressive and anxious symptoms [76]. Interventions in this domain are very difficult to implement successfully. As evidenced by current trends, large-scale policy change accompanied by compliance and willingness from local authorities is needed to improve housing conditions and tackle discrimination. Programmes and policies to address the discrimination that these communities face exist in most countries, but a report from 2015 found that their implementation was widely ineffective [77]. It found that marginalisation during policy formulation, lack of funding, and poverty all work to limit Roma representation in government [77]. Properly implementing these policies and removing barriers to Roma representation in policy-making, may prove to be very effective at combatting stereotypes and reducing discrimination. Increasing access to education and reducing unemployment should be priorities, as these will allow for vertical mobility of Romas within the respective societies that they reside in. In qualitative interviews, participants stated that employing more Travellers in health services targeted toward Travellers would help increase trust in these services. They mentioned this could also include more Travellers in non-clinical roles, such as receptionists [59]. The knowledge that their own people are working as representatives of a service would help them feel more at ease in engaging with it. This may work on a multifactorial level to not only increase engagement with settled services, but also decrease unemployment and help lift these communities from poverty.

#### Physical health and access to healthcare

Another consequence of the poor socioeconomic conditions discussed in the previous section is their impact on healthcare. Romas have significantly worse physical and mental health outcomes [17]. This disparity was clearly elucidated by Watkinson et al. and Hayanga et al. [20, 33]. Romas report higher levels of several chronic health conditions, including cardiovascular disease, arthritis, and diabetes, and they are the likeliest to have multiple long-term conditions [20, 22, 23, 27]. Chronic illnesses are associated with higher rates of mental health problems, largely due to the psychological impact of having a chronic condition [78]. In some cases (such as arthritis) common pathophysiological processes also increase the risk of mental health disorders such as depression [79]. This can make the condition harder to treat, creating a loop where mental and physical health worsen each other [80]. Furthermore, in Zelko et al., physical symptoms that Romas faced were associated with higher levels of mental health diagnoses, including mobility issues, chronic pain, and inability to partake in everyday activities [50]. What results is a bidirectional link between physical illness and mental health concerns; both exacerbate each other and lead to a greater and more severe burden on the patient [79]. Therefore, poor physical health status of Romas not only negatively affects their mental health status, but it also creates a vicious cycle by which physical and mental symptoms both get worse.

Romas in both the EU and the UK have lower access to and utilisation of healthcare services than the general population [28, 81]. Roma and Traveller culture tends to be fatalist and stoic in relation to its perspective on health; it is widely believed that ill health is a normal part of life, and nothing can be done about it [82]. As mentioned before, there is also a sense of alienation from “settled” institutions; studies have shown that Romas fear hostility and prejudice from healthcare providers, making them less likely to turn to them [17]. Romas have faced discrimination within healthcare; for example, forced sterilisation of Romas was widespread in Europe until the early 1990s [81]. In Roma culture, a great deal of importance is put on the “collective”, or the immediate Roma community that one resides within. A Swedish study found that Roma women almost always presented to primary care as a small group of patients all experiencing the same symptoms and wanting the same treatment [83]. They found comfort in this group setting, as it gave the women the support of a community whose approval is needed to seek the help they need [83]. Stigma surrounding mental health is even more pervasive in Roma and Traveller communities, with mental health very rarely being discussed openly [84]. This stigma makes it very hard for Roma and Traveller individuals to access care for mental ill health, with men facing additional barriers due to gender norms [84]. Most Travellers who were offered group psychotherapy at one community mental health team in Ireland declined this treatment option [46]. The examples in this paragraph show that the collective’s opinion, whether that of validation or stigma, has an impact on health and help-seeking behaviours [83].

The example regarding group psychotherapy also shows that members of these communities are likelier to forego utilising healthcare services at all if these services force them to face a group of other Travellers in a vulnerable, “ill” state [46]. However, it is interesting to note that in Ireland, studies show Travellers utilise healthcare services more than the general population, fitting with their poorer health status [82]. Rather than a difference between Irish Travellers and GRTs in the UK, this seems to be due to a difference in the Irish and UK healthcare systems. This is evidenced by the fact that Travellers in the Republic of Ireland utilise healthcare services significantly more than those in the UK province of Northern Ireland, which falls has the same healthcare system as the rest of the UK [82]. This may be due to the success Ireland has had in implementing its “medical card” programme within the Traveller community. This programme allows low-income individuals to access healthcare services free of charge, and also allows them to do so anywhere in the country—suiting those maintaining a nomadic lifestyle [85]. 92.6% of Travellers had a medical card, which reduces financial barriers, as well as other barriers to healthcare by providing them with a physical symbol of entitlement to access healthcare services like any other Irish citizen [19]. Additionally, there are “Primary Health Care for Traveller Projects” across Ireland, which are partnerships between the Irish government’s healthcare service and Traveller organisations [7]. They work to train Travellers to become community health peer workers, essentially resulting in culturally competent health support [7]. Although similar programmes exist in the UK, they are not standardised throughout the country [86, 87]. They also have no evidence of working in partnership with Roma/Traveller organisations to inform their care, which is perceived to be crucial by Travellers [59].

The disparity highlighted above between Travellers in Ireland and Romas/Travellers in the UK show that despite the power of stigma, improving access to healthcare services may improve their utilisation rates as well. General practices often require a permanent address for registration, which many Romas/Travellers do not have. In the UK, where there is no “medical card” system and very fragmented programmes targeted at Roma/Traveller communities, this makes it near impossible to access primary care [87]. This is especially true considering these communities are caught in a cycle of unauthorised camping and forced evictions. There is a heavy reliance on walk-in centres and hospital emergency departments, which have no continuity of care or follow-up capabilities; this can lead to interruptions in treatment and leads to worse health outcomes [17]. Romas/Travellers in the UK use hospital emergency departments significantly more often than the general population, but are far less likely to be registered with a GP [88]. This is in sharp contrast with Ireland, where Travellers still access hospital services more than the general population, but have equal rates of access to primary care [73]. Across Europe, 26% of Romas are not covered by health insurance [89]. There is also a dearth of medical facilities and professionals who work in the remote areas and segregated Roma settlements [81]. These factors make it much more challenging for Romas who need mental healthcare to access it. Issues that can be treated are thereby allowed to remain unresolved and worsen, further compounding the negative mental health status of this community. Rees et al. found that those Romas and Travellers in Wales who were registered with a GP utilised mental healthcare resource at a higher rate than non-Romas [43]. This suggests that Romas/Travellers who are given access to mental healthcare do utilise it and improving this access would result in more Romas and Travellers getting the care they need [43].

However, the same study found that although attendance rates at first psychiatric appointments were the same between Romas/Travellers and the general population, follow-up attendance was significantly lower in the Roma/Traveller population [43]. Based on this, more needs to be done to emphasise the importance of continuity of care in this community, so that mental health professionals can maximise the impact of their care. Other interventions in this area should focus on improving education on mental health issues to destigmatise this topic. Roma health mediators could have a huge impact in this area. These are Roma individuals trained to act as liaisons between the Roma community and the healthcare system. In countries that have Roma health mediator programmes, there has been increased education about health and vaccination rates in the Roma community, among many other benefits [90]. Using these programmes to target mental health could be a targeted, effective way to improve outcomes. The Irish Primary Health Care for Traveller programmes are an example of an intervention that trains Travellers to become ambassadors of healthcare within their communities [7]. Traveller Mental Health Liaison Nurses have also been used in Ireland and have been received extremely well by the community [56]. Attributes that raised trust in these services included privacy, cultural competence, emphasis on holistic methods of maintaining well-being, and the ability to signpost to mainstream social/healthcare services [56]. It is crucial that these interventions are standardised across countries and work equally alongside organisations that represent these communities to inform their activities. Lopez et al. used a church organisation to promote a safe space where issues surrounding substance abuse and mental health could be explored and solutions discussed [54]. Using respected community institutions where individuals spend a significant amount of socialising time may help to reduce the shame and guilt associated with issues such as mental health [54]. Additionally, improving access to primary care is essential, as this would improve continuity of care and reduce reliance on hospital emergency department and walk-in centres. Primary care physicians should be given information on how to register and care for nomadic people. A programme similar to the Irish medical card programme, but specifically tailored to allow people with no fixed address to access GP appointments, may enhance primary care services for Romas and Travellers [85].

#### Women’s mental health

Evidence suggests that Roma/Traveller women face a proportionately higher burden of mental health issues when compared to men, and that they face additional challenges that are not faced by Roma/Traveller men [50]. These challenges are multifactorial, but largely revolve around the strict gender roles and norms that are embedded in Roma and Traveller culture. The resulting system is patriarchal and can have a profound impact on women’s everyday life, and by extension, their health [9].

Roma/Traveller boys and girls are both often removed from formal schooling very early [91]. However, Roma girls are removed earlier than boys, and the two genders also go down very different paths [91]. Boys tend to join their fathers at work, while girls are typically expected to stay at home with their mothers [92]. In Roma/Traveller culture, this occurs at the time of menarche; from this age they are expected to help with household duties and take care of younger children [12]. Rigid rules of etiquette are expected of women, and deviation from these rules can bring dishonour to them and their family [12]. These values are strictly enforced, and girls are raised to internalise them, regardless of their own ambitions [12]. From childhood, to adolescence, and into adulthood, many of these women are unable to make decisions regarding their own lives. The majority of Roma and Traveller women are inactive from the job market and are financially reliant on the men who hold authority over them [91]. This creates a power structure where women’s autonomy is suppressed. Self-determination theory states that autonomy is vital to an individual’s ability to function, and a lack of autonomy can affect psychological well-being negatively [93]. Controlled motivation to make decisions is associated with worse mental health outcomes. Meanwhile, autonomous motivation leads to not only better mental health, but also long-term changes towards more positive, healthy behaviours [93]. Therefore, this lack of autonomy may to a large degree be driving the worse mental health outcomes that Roma women experience. Additionally, Traveller and Roma women may be more recognisable as Travellers/Romas than males due to traditional dress codes. This makes them more prone to discrimination and, by extension, the negative impacts on mental health that stem from this [94].

Roma and Traveller women also tend to marry at a much earlier age. Inel found that 59.6% of Roma women studied were married before the age of 18 [10]. Other sources from various countries support the notion that Roma girls are much more likely to marry before the age of 18 [95, 96]. The 2016 Irish Census found that 31.9% of Travellers aged 15–29 were married, compared to 5.8% of the same age group in the general population [74]. Early marriage is associated with higher rates of depression and anxiety, with child marriage also being a risk factor for suicidal ideation/suicide attempts [97, 98]. In fact, Inel found that in women who were married as children, the length of the marriage was positively associated with depressive, anxious, paranoid, psychotic, and somatisation symptoms [35]. Besides somatisation, none of these symptoms were associated with length of marriage in women who married after they turned 18, suggesting that the age of marriage has a profound impact on women’s mental health status [99]. Filakovska et al. found that Roma mothers face a higher level of stress than non-Roma mothers, and this is associated with harsher forms of disciplining children [32]. The study went on to say that these disciplinary actions were associated with negative mental health outcomes in children, showing how these issues are multi-generational and can affect not only the woman’s mental health, but also her child’s [32].

A third issue disproportionately faced by women is domestic violence. Although it is very difficult to find statistics on the matter, foundations that work with these communities report elevated rates of domestic violence [92, 94]. They also face more barriers than the general population in speaking out about this violence and stopping it from happening. These include illiteracy, lack of education, distrust of authorities, and widespread normalisation of abuse [17]. Both male and female Romas are more likely to find domestic violence justifiable. Across the EU, 30% of Roma women and 34% of Roma men think it is reasonable for a husband to slap his wife, compared to 15% of non-Roma women and 17% of non-Roma men [91]. Kozubik et al. showed that violence is often downplayed by the perpetrator, the victim, and the family [13]. It is commonly implied that these incidents are meant to happen in healthy marriages, or that the incident was not as bad as it seems [13]. Common reasons for not seeking help were financial dependence on the abuser, the belief that the abuser would change, and the lack of help or support from family [13]. A report from the Traveller foundation Pavee Point also highlighted that Traveller women are reluctant to speak out, for fear of reinforcing negative stereotypes that exist about the Traveller community [94]. Domestic violence has a significant impact on mental health. It is known to increase rates of depression, anxiety, post-traumatic stress disorder, suicidal ideation, and substance abuse disorders, as well as exacerbate psychotic symptoms [100, 101]. Kozubik et al. corroborates this point, showing that 75% of the survivors surveyed had psychological concerns, 25% having anxiety or depression [13]. Domestic violence, and being trapped in an abusive marriage, therefore forms another key source of mental health issues for Roma and Traveller women.

Women from these communities can often become trapped in a very isolated space, where it becomes difficult to speak up about issues they may be facing. Interventions to better Roma/Traveller women’s mental health should therefore focus on creating a space where Roma and Traveller women feel safe disclosing information to professionals who can then provide them with next steps. Efforts should also be made to build back the autonomy of Roma/Traveller women, by improving education and fostering initiatives which enable them to make their own decisions. An example of this is the RoMoMatteR project, which aimed to develop critical thinking skills in Roma girls and allow them to make their own decisions about their future while maintaining the values important to them [102]. The results of the project suggest that the intervention was effective. Roma girls were able to develop solutions to problems that arose in their life and build capacity to make their own decisions [103]. Educational interventions like these may also be better received by the Roma/Traveller community. Major campaigns to end child marriage, gender discrimination, and domestic violence in these communities can become politicised and used to fuel further discrimination.

#### Suicide

Zonda et al. showed that the Roma population reported far more suicide attempts than the non-Romas [51]. However, the study also found that despite attempting suicide more often, Romas completed suicide far less frequently than the general population [51]. Despite this evidence, a more recent UK study completed in 2024 shows that Gypsies and Irish Travellers have the highest death-by-suicide rates of any ethnicity [62]. The study showed that both males and females from these communities had higher rates of suicide than the majority (White British) population, with GT females having a suicide rate that is more than twice that of White British females [62]. Given that Zonda et al. was carried out in 1990, these new findings may indicate a change over time indicating that suggests suicidality is becoming more common and serious in these communities. It may also suggest differences in suicidal behaviour between the Romas of Europe and the UK. The study did not examine rates of attempted suicide in addition to death by suicide, so it is not possible to tell whether the GT community’s attempted suicide rate is higher than that of the majority White British population [62].

Multiple studies comparing self-harm, suicidal ideation, and suicidal behaviour have found Travellers to be significantly likelier to self-harm, as well as attempt and die by suicide, than the general Irish population [31, 36, 45]. Travellers in the age group of 20–29 have the highest rates of self-harm and suicidal ideation presentations to emergency departments of any ethnicity in Ireland [36]. Keogh et al. found that all 10 Travellers interviewed had lost family members or close friends to suicide, with 2 participants having attempted suicide and a third having expressed suicidal ideation [53]. The all-Ireland Traveller Health Study found that in 2008, 11.2% of all Traveller deaths were by suicide. The male Traveller suicide rate in particular was 6.6 times higher than that of the general Irish male population [19].

Romas appear to attempt suicide for different reasons than the general population [51]. Toth et al. showed that the majority of Romas did not have the intent to die; instead, most of them attempted suicide to “escape from an unbearable situation” [47]. They were far likelier than the general population to attempt suicide impulsively without planning the attempt [47]. Additionally, despite similar levels of depression and hopelessness in Romas and non-Romas, the Roma subjects were likelier to attempt suicide multiple times [47]. Similar conclusions were reached in Zonda et al., where a common reason for Romas’ suicide attempts was to “appeal to others” [47]. This may suggest a difference in perspective on suicide between Roma communities and the general population. Toth et al. suggests that for Romas, suicide may not simply be the act of ending one’s life. Rather, it may be a way for Romas to display the gravity of their individual circumstances. By deviating from the simple conflation of suicide with death, Romas may be unconsciously allowing the act of attempting suicide to become a legitimate way to express their distress. This is especially plausible considering the stigma around mental health and the stoicism that is prevalent in Roma and Traveller cultures.

Smoking, long-term unemployment, and a family history of suicide are all factors that are associated with an elevated risk of attempted suicide in the Romas, but not in the non-Roma population [47]. Smoking is an example of an emotion-based coping strategy. Individuals use smoking to help regulate negative emotions rather than focusing on the problem causing these emotions [104]. Long-term unemployment is an example of a common problem that can affect mental health, especially in deprived Roma and Traveller communities [47, 53]. A family history of suicide can normalise the act of taking one’s own life and make it easier for an individual to turn to this as a legitimate behaviour. These findings provide further evidence that suicide may be used as a more extreme form of emotion-based coping in Roma populations rather than a definitive end of life.

Significant stigma exists around the topic of suicide in Roma and Traveller communities [47, 55]. Suicidal ideation and behaviour are often seen as topics that are too shameful or sensitive to talk about, which becomes a barrier to those seeking help [105]. Individuals who are suffering become left behind to sort out their own problems. Engaging family in the patient’s care after a suicide attempt is a common and important practice that helps to prevent recurrent suicide attempts [106]. However, Kavalidou et al. found that Travellers who presented to emergency departments with self-harm/suicidal ideation or self-harm acts were significantly likelier to request no next-of-kin involvement compared to the general population [36]. This shows that not only does stigma around suicide worsen mental health to the point where it becomes a viable option. It can also interfere with important strategies that mental health services rely on to reduce recurrence of suicidal behaviour. As with mental health, this stigma is worse for men, who are expected to be constantly mentally strong [58].

Interventions to reduce suicidality in Roma and Traveller communities should focus on destigmatising suicide and reframing it as an important and preventable issue. Malone et al. implemented a joint science-arts-based intervention. Interviews with Travellers who had been affected by suicide were interspersed between art projects relevant to suicide in the Traveller community, creating an exhibition that explored the topic in depth. The authors emphasise that the inclusion of Travellers in the planning of the project was essential to its success and allowed it to reach a community that is otherwise hard to engage in conversation. Community-based interventions can provide a setting to “break the ice” about unspoken issues, where Romas and Travellers feel safe with their own people. When Roma/Traveller families and communities can talk openly about suicide, it will allow for community members to pick up early signs of suicidality in individuals who can then be helped rather than shamed [55]. Given the protective nature of familial support in these communities and the reluctance seen in Travellers to involve family after a suicide attempt, family-based interventions should also be explored [47]. Allowing Romas and Travellers to feel safe to involve their family in post-suicide attempt care could also specifically help to reduce multiple suicide attempts, which was found to be a concern in these communities [47]. Services aimed at destigmatising suicide should follow the same important criteria attributed to success in Sect. " Physical Health and Access to Healthcare" to create a trusting and open relationship between patients, families/communities, and service providers. Interventions suggested in other sections of this review, which aim to improve Roma/Traveller mental health, would also help to lower suicidality. This is because they prevent mental health issues from getting to the point where suicide seems like the only option.

### Strengths and limitations

This review has several strengths. A broad search of all OVID databases allowed for collection of studies not only from medical journals, but also from applied health sciences and social sciences journals to get a more holistic view of the topic. Additionally, studies were included from a large variety of countries in Europe. As Roma culture and communities can vary largely by region, this allowed the issue to be considered from a broader perspective, to determine whether there were common themes throughout the areas where they reside.

However, the review is not without its limitations. Firstly, no quality appraisal checklist was available for mixed-methods studies. For these studies, the specific results that were most relevant to the research question were analysed to determine the methods that allowed the researchers to obtain them. The appropriate checklist was then utilised. Another limitation of the paper is the decision to exclude non-English language studies. However, we do not believe this to be a major limitation. Although 8 non-English articles were identified by the electronic literature search in total, only 2 of these discussed Roma or Traveller mental health, with 1 being a case study that would have been excluded from this systematic review.

A meta-analysis was not conducted because there was too much heterogeneity within the included sample of studies. Meta-analyses require a sharper focus so that quantitative results from studies can be pooled together to establish definitive conclusions on a specific question. The evidence in this review explored various topics, and did so both qualitatively and quantitatively, making the review too broad in its scope for a meta-analysis to be appropriate [107].

The evidence base included in the review also has several limitations. One area that could be improved in many studies was the sampling process. Many studies drew Roma and Traveller subjects from one or two areas of their country. For example, Gouva et al. used two prefectures in Southern Greece to recruit all their Roma subjects, whereas Heredia-Amador et al. used only the rural Spanish city of Guadix [34, 60]. Similarly, Tong et al. used the database of one community mental health team, and Tanner et al. used one tertiary hospital to draw results regarding Travellers [45, 46]. As a result, their results and the conclusions that were drawn may not be generalisable, as they may only describe phenomena that are present within these specific areas. This is especially true for Heredia-Amador et al., as there are significant differences in urban and rural life, which could have a large impact on the results found [34, 60]. Additionally, many studies seemed to define someone’s ethnicity solely through self-reporting. This may lead to discrepancies, as Roma and Traveller people are known to sometimes hide their ethnicity on paperwork and documents to avoid discrimination. In these studies, it is unclear whether actions were taken to avoid this from happening [47, 49]. A study by Rees et al. was also assessed to have an issue with the sample frame. As they recruited Roma subjects based on geographical location data and GP records, they limited their study of Roma mental health to those Romas who were living on an authorised site and were registered with a GP [43]. Not including Romas on unauthorised sites or those without access to a regular GP could markedly change the results. However, it should be noted that recruiting Roma subjects, who can be isolated from “settled” society, often do not engage with or access “settled” society’s institutions, and can be distrustful of outsiders, is a daunting task. Therefore, the sampling process that these studies chose may have been the only practical option. An issue that was seen specifically in Kolarcik et al. was that Roma children were given qualitative interviews due to concerns around illiteracy, but non-Roma children were given written surveys. The study’s authors themselves note that interviews are associated with more positive responses, and this may have led to underestimations in the health differences between these two communities [37]. A similar issue was seen in Vazsonyi et al., where Roma adolescents were given pencil-and-paper surveys, but non-Roma adolescents were given online surveys [48]. Lastly, most studies were prevalence studies and analytical cross-sectional studies. According to the hierarchy of evidence, although these still offer an invaluable insight, their evidence is weaker than that of case–control studies, cohort studies, and randomised controlled trials, as they cannot establish causation [108].

It was also quite difficult to write about controversial issues such as women’s rights within the Roma and Traveller communities without seeming to make pejorative inferences about the culture and traditional ways of these communities. However, it is important not to dismiss any possible mechanisms for the mental health outcomes discovered in this review.

Lastly, studies investigating suicide require very large sample sizes with long follow-up periods to enable researchers to conduct analyses with adequate statistical power. Roma and Traveller populations tend to be fragmented, small, and possibly nomadic, which makes it hard for studies meeting these criteria to be conducted. Individuals are also often not documented in information systems and databases such as censuses. This means the conclusions from these studies may be weaker, as they may not include all suicide (both attempted and completed) that have occurred in the Roma community. Zonda et al. mitigated this by following up with the vast majority of their sample [51].

### Further research

As stated above, much of the research was quantitatively descriptive (prevalence estimation), cross-sectional, or qualitative. Further research should focus on higher quality research, including cohort studies with long-term follow-up, and randomised clinical trials. These would allow for causation to be established and clarify the relationship that the factors explored in this review have on mental health.

Further research should also focus on the mental health of children, as such studies included in this review had varying findings [37, 48]. In future studies on this topic, special care should be taken to avoid differences in the delivery of surveys between Romas/Travellers and non-Romas/Travellers, as these may have had a large impact on the studies’ findings and contributed to the contradictory nature of the results.

Research regarding interventions should be prioritised as well; extremely limited research regarding this was found in this review. Those interventions that did exist had the potential to be replicated on a larger scale and should be researched further. The efficacy of Roma and Traveller health mediators for physical health has been well-documented, and more research on targeted Roma/Traveller health mediators for mental health such as that in O’Sullivan et al. should be explored [56].

## Conclusion

In conclusion, this systematic review shows that Romas and Travellers face significantly worse mental health outcomes and a higher risk of suicide when compared to the general population. Contributing factors to this trend are multifactorial, encompassing socioeconomic deprivation, disparities in physical health, and low access to healthcare. Interventions will need to be comprehensive to effect positive systemic change in these communities. Due to attitudes in these communities around healthcare and mainstream society, community-based interventions targeted to Roma/Traveller culture are needed. The evidence was weakened by the lack of randomised controlled trials and cohort studies, with most of the studies being descriptive, cross-sectional, or qualitative. However, inclusion of qualitative studies provided a unique insight into the effects of negative mental health on the lives of Roma/Traveller individuals and what they felt was important in the development of mental illnesses and the interventions that should be implemented. This Roma/Traveller perspective is something that is often missing from interventions, but vital to ensure their success. Further research should focus on improving the evidence base, exploring children’s and adolescent’s mental health, and studying the quality of interventions.

## Supplementary Information

Additional file 1. Supplementary Table 1: Key information from each study included in the review, including author, country, year, study design, title, sample size, Roma in sample, mental health measurement, and key findings.

Additional file 2. The JBI Quality Appraisal Checklists for each study included in the review, organized in alphabetical order.

## Data Availability

All data analysed during this study are included in this published article and its supplementary information files.

## Abbreviations

GRT: Gypsies, Romas, and Travellers, the designation used for these communities on most UK government resources/documents
MeSH: Medical Subject Heading
GT: Gypsies and Travellers, designating that the study being discussed is UK-based and looked at British Gypsies/Irish Travellers, not Romas who are considered by the UK Government to be more recent immigrants from Europe with less roots in the UK
PSS-4: Perceived Stress Scale
CREDI: Caregiver Reported Early Development Instruments
ESS: Experiences of Shame Scale
OAS: Other As Shamer
STAI: State-Trait Anxiety Inventory
HADS: Hospital Anxiety and Depression Scale
EQ5D: Euroqol-5D
GPPS: General Practitioner Patient Survey
GDS-EV: Geriatric Depression Scale
BSI: Brief Symptom Inventory
ASR: Age Standardised Rate
IRR: Incidence Rate Ratio
SDQ: Strength and Difficulties Questionnaire
CELS: Communicative Everyday Life Stories
LS: Single-Item Satisfaction Scale
IPA: Interpretative Phenomenological Analysis
BDI: Beck Depression Inventory
IPDE: International Personality Disorder Examination
