# Supplementary material for: Mental illness and suicidality among Roma and traveller communities in the UK, Ireland, and other countries: a systematic review
Source: BMC Psychiatry. 2025 Apr 4;25:331. doi: 10.1186/s12888-025-06752-0 (PMC11969765; doi:10.1186/s12888-025-06752-0)
Supplement: Supplementary file 1 — Additional file 1. Supplementary Table 1: Key information from each study included in the review, including author, country, year, study design, title, sample size, Roma in sample, mental health measurement, and key findings. [file 12888_2025_6752_MOESM1_ESM.docx]

Supplementary Table 1. Key information from each of the studies included in the review.

| **Author, Country** | **Year** | **Study Design** | **Title** | **Sample + Roma in Sample** | **Mental Health Measurement**^a^ | **Relevant Key Findings** |
| --- | --- | --- | --- | --- | --- | --- |
| Abdalla et al. (1)  ***Ireland*** | 2013 | Analytical cross-sectional | Disparities in fatal and non-fatal injuries between Irish Travellers and the Irish general population are similar to those of other indigenous minorities: A cross-sectional population-based comparative study | 4956 | Deaths/injuries coded as being intentional or due to mental or behavioural disorder (according to the ICD-10 coding system) in the Irish General Registrar Office’s official database. | Travellers are significantly likelier to die of intentional injury (Standardised Mortality Ratio of 637 in men and 434 in women when compared to the general population).  Travellers are significantly likelier to inflict intentional injury on themselves (Standardised Incidence Ratio of 181 in men and 268 in women when compared to the general population). |
|  |  |  |  | 1771 Irish Travellers^b^ |  |  |
| Barrio-Forne et al. (2)  ***Portugal*** | 2021 | Case-control | Companionship as a method to reduce anxiety in pregnant women hospitalized during their third trimester | 80 | **Anxiety**: State-Trait Anxiety Inventory tool | Women of the Roma ethnicity are more likely to develop anxiety during pregnancy (*p=0.019)* |
|  |  |  |  | 4 Roma |  |  |
| Carrasco-Garrido et al. (3)  ***Spain*** | 2011 | Analytical cross-sectional | Health Status of Roma Women in Spain | 1581 | National health surveys in Romani population and general population were used, had sections for reporting conditions | 21.4% of Roma women experienced depression or anxiety, compared to 14.2% of non-Roma women.  Roma women are more likely to drink alcohol (35.1% Roma vs. 16.5% non-Roma).  Roma women are more likely to develop obesity (24.45% vs. 10.22%) and migraines (37.76% vs. 14.42%).  Roma women report a poorer self-perception of their health than non-Roma women. |
|  |  |  |  | 527 Roma |  |  |
| Filakovska et al. (4)  ***Slovakia*** | 2014 | Analytical cross-sectional | Perceived Stress of Mothers, Harsh Discipline, and Early Childhood Mental Health: Insights from a Cross-Sectional Study in Marginalized Roma Communities | 173 | **Perceived stress:** Shortened Perceived Stress Scale (PSS-4)  **Childhood mental health:** Mental Health subscale of Caregiver Reported Early Development Instrument (CREDI, higher = worse health) | Roma mothers have more perceived stress than non-Roma mothers (9.54 vs. 7.85 on PSS-4 where higher = more stress, *p <0.001)*.  Roma mothers discipline children harsher than non-Roma mothers *(p <0.001).*  Roma children have worse mental health than non-Roma children (4.43 vs. 2.24 on CREDI, *p <0.001)*. |
|  |  |  |  | 94 Roma |  |  |

| Gouva et al. (5)  ***Greece*** | 2015 | Prevalence | Shame and Anxiety Feelings of a Roma Population in Greece | 194 (all Roma) | **Shame:** Experiences of Shame Scale (ESS) & Other As Shamer (OAS)  **Anxiety:** State-Trait Anxiety Inventory (STAI) | Roma people, both men and women, experience high levels of shame and anxiety. Higher internal and external shame are associated with higher trait anxiety; higher chance of having anxious personality *(p<0.05)*.  Women experience higher levels of internal shame (ESS scores 52.27 vs. 45.42, *p<0.001*), whereas men experience higher levels of external shame (OAS scores 35.93 vs. 30.87, *p<0.05*).  Women are likelier to have high state anxiety than men; likelier to show anxiety in stressful situations (STAI score 48.83 vs. 43.20, *p <0.001*). |
| --- | --- | --- | --- | --- | --- | --- |
| Goward et al. (6)  ***UK*** | 2008 | Mixed-methods: prevalence & qualitative | Crossing boundaries: identifying and meeting the mental health needs of Gypsies and Travellers | Prevalence: 60 Gypsies/Irish Travellers (GT)^b^ | **Anxiety/Depression:** Hospital Anxiety and Depression Scale (HADS)  **Health-Related Quality of Life:** Euroqol 5D (EQ5D) | GTs had significantly higher anxiety/depression scores (HADS scores 15.4 vs. 9.8), and significantly lower health-related quality of life (EQ5D scores 0.62 vs. 0.86)  Higher proportions of GTs had anxiety (36.7% vs. 12.6%) and depression (26.7% vs. 3.6%), defined as a score of 11+ on that subsection of HADS.  Based on qualitative research, financial difficulties, loss of loved ones, and housing/physical environment were seen as reasons for low mood. Family support was a protective factor.  Based on qualitative research, healthcare providers experience many difficulties in trying to resolve GT mental health concerns. These include lack of access/coordination/engagement, nomadism compromising continuity of care, and a lack of knowledge about the community’s specific needs. |
|  |  |  |  | Qualitative:  17 (all GT)^b^ |  |  |
| Hayanga et al. (7)  ***UK*** | 2023 | Analytical cross-sectional | Ethnic inequalities in age-related patterns of multiple long-term conditions in England: Analysis of primary care and nationally representative survey data | 1 432 641 | Data used from the GP Patient Survey (GPPS), a large-scale postal survey asking patients questions about their health and demographics. | Compared with any other ethnic group, GTs have the highest prevalence of a long-term mental health condition (14.9%, with the next highest being 7.9% in White/Asian mixed people).  Compared with any other ethnic group, GTs have the highest prevalence of multiple long-term conditions, including mental health conditions (9.9%, with the next highest being 6.2% in White/Black-Caribbean mixed people). |
|  |  |  |  | 365 Gypsies/Irish Travellers (GT)^b^ |  |  |
| Heredia-Amador et al. (8)  ***Spain*** | 2018 | Analytical cross-sectional | Differences in the prevalence of depression in older Spanish Romany and non-Romany people and associated factors | 181 | **Depression:** shortened version of the Geriatric Depression Scale (GDS-EV) | A larger proportion of the Roma men had depression compared to the non-Roma men (17.78% vs. 2.27%, *p<0.001*).  A larger proportion of the Roma women had probable depression or depression compared to the non-Roma women (64.00% vs. 28.52%, *p<0.001*).  Higher GDS-EV scores were positively associated with age, number of children, number of medicines, and dependence on others (*p<0.05* for all variables). They were negatively associated with education, exercise, visits from family, and feeling valued by family (*p<0.05* for all variables). |
|  |  |  |  | 95 Roma |  |  |
| Inel (9)  ***Turkey*** | 2024 | Analytical cross-sectional | An evaluation of early marriage and the mental state of Roma women: A cross-sectional study | 272 (all Roma) | **Psychological symptomology:** Brief Symptom Inventory (BSI) | 59.6% of the women were married before the age of 18, and these women had higher rates of chronic physical health conditions (48.8% vs. 28.8%, *p<0.001*).  In women ≤18 at marriage, longer duration of marriage was significantly associated with somatization, depression, anxiety, paranoid thoughts, psychoticism. In women >18 at marriage, duration of marriage was only significantly associated with somatization. |
| Kavalidou et al. (10)  ***Ireland*** | 2023 | Analytical cross-sectional | Presentations of self-harm and suicide-related ideation among the Irish Traveller indigenous population to hospital emergency departments: Evidence from the National Clinical Programme for self-harm | 24 473 | Presentations to the emergency department recorded in the National Clinical Programme for Self-Harm and Suicide-Related Ideation by a nurse/doctor as being a “self-harm act”, “suicidal ideation”, or “self-harm ideation”. | Female Traveller patients were 3.04 (95% CI 2.51-3.68) times likelier to present with suicidal ideation and had a rate of self-harm presentations which was 3.85 (95% CI 3.35-4.41) times higher compared to female White Irish patients.  Male Traveller patients were 4.46 (95% CI 3.86-5.16) times likelier to present with suicidal ideation and had a rate of self-harm presentations 5.43 (95% CI 4.75-6.21) times higher than White Irish patients.  Male Travellers aged 20-29 have the highest rates of ED presentations related to self-harm and suicidal ideation of any age band for any ethnicity in Ireland.  Travellers were significantly likelier to request no next-of-kin involvement after a presentation fitting the 3 categories listed (19% White Irish vs. 28% Irish Traveller, *p<0.001*) |
|  |  |  |  | 744 Irish Travellers^b^ |  |  |

| Keogh et al. (11)  ***Ireland*** | 2020 | Qualitative | Evaluation of a Traveller Mental Health Liaison Nurse: Service user perspectives | 10 (all Irish Travellers)^b^ | Qualitative interviews were used. | Accommodation and financial worries were found to be strongly associated with Traveller mental health. Many participants with poor mental health attributed this to living on halting sites or in rented accommodation with unsuitable conditions.  Stoicism in male Travellers (resulting from traditional gender structures) and public humiliation of those who shared vulnerability, were seen as contributing to mental distress in male Travellers.  All participants had a family member/close friend lost to suicide. 2 participants had attempted suicide & one had expressed suicidal ideation.  Discrimination against Travellers is perceived to be worsening and encroaching on necessary facets of life such as accommodation and employment.  No negative or neutral comments were made about the service; only positive. Trust, confidentiality, and privacy were said to be the most important parts of a service they felt they could engage with. Liaison interventions, where the service was able to signpost users to another “settled” service that could help, were considered very important. |
| --- | --- | --- | --- | --- | --- | --- |
| Knipe et al. (12)  ***England & Wales*** | 2024 | Cohort | Ethnicity and suicide in England and Wales: a national linked cohort study | 31 644 | Used data from Public Health Research Database & 2011 Census to create Age Standardised Rate (ASR) and Incidence Rate Ratio (IRR) compared to the majority White British population. | GT males had an IRR of 1.40, and GT females had an IRR of 2.26. This was the highest IRR of any ethnicity. Thus, GT females had more than double the rate of suicide of females in the White British majority.  GT was the only non-mixed ethnicity to have a higher suicide rate than the White British majority. |
|  |  |  |  | 45 Gypsy/Irish Traveller (GT)^b^ |  |  |
| Kohler et al. (13)  ***Bulgaria*** | 2011 | Cohort | Ethnic and Religious Differentials in Bulgarian Mortality, 1993-98 | Entire 1992 Bulgarian census was used. | **Suicide:** Bulgarian death records; measured in death rate per 1 000 people. | Christian Romas had highest death rate by suicide of any ethnicity. They showed a similar suicide rate to Christian Bulgarians.  Muslim Romas had significantly lower death rates by suicide than Christian Romas. This was the only cause of death where religious differences outweighed ethnic differences. |
| Kolarcik et al. (14)  ***Slovakia*** | 2009 | Analytical cross-sectional | To what extent does socioeconomic status explain differences in health between Roma and non-Roma adolescents in Slovakia? | 1052 | **Anxiety:** subjects were asked if they suffered from anxiety in the last month  **General Mental Health:** Strength and Difficulties Questionnaire (SDQ) | Roma children did not report more mental health difficulties than non-Roma children; 23.8% of Romas reported high difficulties & problems, as opposed to 22.4% of non-Romas.  Study notes that Roma children were given interviews to combat illiteracy, but non-Romas were given written surveys. |
|  |  |  |  | 330 Roma |  |  |
| Kozubik et al. (15)  ***Slovakia*** | 2020 | Qualitative | Health Risks Related to Domestic Violence against Roma Women | 20 (all Roma) | Interviews were done which asked about the effects of domestic violence; these effects included “general” psychological symptoms, as well as anxiety & depression. | Of the 20 women who experienced domestic violence, 75% had general psychological problems, with 25% further reporting anxiety or depression.  Most of the women perceived addiction to be a trigger for violence; this was usually alcohol or gambling. Downplaying of violence was seen as a common strategy used by the husbands to placate the abused women. |
| Lee et al. (16)  ***Romania & Bulgaria*** | 2014 | Analytical cross-sectional | Mental health disparities between Roma and non-Roma children in Romania and Bulgaria | 2372 | **Children’s Report:** Dominic Interactive  **Parent/Teacher’s Report:** Strength and Difficulties Questionnaire | Romas had higher child-reported odds of internalizing disorders (OR = 2.99); phobias (OR = 4.84), separation anxiety disorder (OR = 2.54), generalized anxiety disorder (OR = 2.95), depression (OR = 3.86). Romas had higher child-reported odds of externalizing disorders (OR = 2.84); oppositional defiant disorder (OR = 3.35), ADHD (OR = 2.35), conduct disorder (OR = 3.63). |
|  |  |  |  | 135 Roma |  |  |

| Linehan et al. (17)  ***Ireland*** | 2002 | Analytical cross-sectional | Irish travellers and forensic mental health | 472 | Admissions to Central Mental Hospital from prisons. | Travellers accounted for 3.4% of all forensic psychiatric admissions despite making up just 0.38% of the general adult population. |
| --- | --- | --- | --- | --- | --- | --- |
|  |  |  |  | 28 (all Irish Travellers)^b^ |  |  |
| Lopez et al. (18)  ***Spain*** | 2018 | Qualitative | Drugs and Mental Health Problems among the Roma: Protective Factors Promoted by the Iglesia Evangelica Filadelfia | 8 (all Roma) | Qualitative interviews were used to assess participants’ health and lives, using a “Communicative Everyday Life Stories (CELS)” technique. | The church provided a sense of belonging and promoted discourse about well-being and happiness, which were found to be protective factors for Roma mental health.  Religious institutions such as a community church organization can provide a means to deal with substance abuse issues, as well as a strong support network to talk about and deal with mental health issues. |
| Malone et al. (19)  ***Ireland*** | 2017 | Qualitative | Lived Lives: A Pavee Perspective. An arts-science community intervention around suicide in an indigenous ethnic minority | 79 (all Irish Travellers)^b^ | Qualitative interviews were used. | 90% of Travellers believed that the intervention was an effective way to address suicide in the Traveller community in Ireland.  68% of Travellers believed that the intervention was effective in suicide prevention or effective in providing bereavement following a suicide. |
| McGorrian et al. (20)  ***Ireland*** | 2013 | Analytical cross-sectional | Frequent mental distress (FMD) in Irish travellers: Discrimination and bereavement negatively influence mental health in the All-Ireland Traveller Health Study | 1791 (all Irish Travellers)^b^ | **Frequent mental distress (FMD)** was defined as 14 or more days of self-reported “days of mental ill-health” | 12.9% of Travellers experienced FMD, compared to 5.0% of the general Irish population.  Perceived discrimination and bereavement of family in the last year were the most predictive factors for developing FMD. |

| Olah et al. (21)  ***Hungary*** | 2023 | | Analytical cross-sectional | | Residence in segregated settlements (colonies) rather than Roma identity increases the risk of unfavourable mental health in Hungarian adults | | 811 | | **Psychological Well-Being over 14 days:** WHO Well-being index  **Life Satisfaction:** Single-Item Life Satisfaction Scale (LS)  **Psychological morbidity:** General Health Questionnaire | | | | Romas scored lower on subjective well-being (15.91 vs. 17.20, *p<0.001*), life satisfaction (7.07 vs. 7.63, *p<0.001*) than non-Roma Hungarians.  17.8% of Romas, compared to 10.3% of non-Roma Hungarians, were at high risk of psychological morbidity (*p<0.001*).  The largest negative influence on poor mental health was found to be living in segregated colonies. | |
| --- | --- | --- | --- | --- | --- | --- | --- | --- | --- | --- | --- | --- | --- | --- |
|  |  |  |  |  |  |  | 287 Roma | |  |  |  |  |  |  |
| O’Sullivan et al. (22)  ***Ireland*** | 2021 | | Qualitative | | The role and activities of the Traveller Mental Health Liaison Nurse: Findings from a multi-stakeholder evaluation | | 34 (all Irish Travellers)^b^ | | Qualitative interviews were used. | | | | It was important for a mental health liaison nurse to avoid labelling their job as mental health related. This could be achieved by avoiding a uniform and referring to the service as “well-being” oriented rather than “mental health” oriented.  A key area that the nurse needs to be knowledgeable on to work effectively with Travellers was knowledge of all services available to Travellers and ability to signpost to other services when needed, including more specialist/mainstream mental health services. Providing this link between Travellers and mainstream services was very valuable.  Well-being workshops where interventions centered around relaxation and mindfulness were effective. Mental health education was able to be offered at these workshops by linking it to a holistic view of “well-being” rather than something clinical.  Preventative measures, psychosocial interventions, and an emphasis on self-care were perceived as more effective than using biomedical interventions and clinical outcomes to judge “treatment success” | |
| Parry et al. (23)  ***England*** | 2007 | | Analytical cross-sectional | | Health status of Gypsies and Travellers in England | | 520 | | **Anxiety/Depression:** anxiety or depression dimension of the EQ-5D | | | | GT had higher prevalence of anxiety than non-GT (39% vs. 13%, *p<0.001*). 8% of GT were extremely anxious, compared to 2% of non-GT. This was one of the most marked differences of all the illnesses studied.  GTs with a long-term illness are likelier to be found in council sites or houses compared to private sites or empty land (*p=0.03*). Those who rarely travelled had the worst health in the past year (*p<0.001*), and the worst EQ-5D scores (*p<0.001*). | |
|  |  |  |  |  |  |  | 260 Gypsy/Irish Traveller (GT)^b^ | |  |  |  |  |  |  |
| Quirke et al. (24)  ***Ireland*** | 2022 | | Analytical cross-sectional | | Experience of discrimination and engagement with mental health and other services by Travellers in Ireland: Findings from the All Ireland Traveller Health Study (AITHS) | | 6450 (all Irish Travellers)^b^ | | Survey questions asking whether access to and experience of mental healthcare services for Travellers is better, worse, or the same as the general population. | | | | Travellers who felt they had worse access to mental health services were 1.83 times likelier to report experiences of discrimination (*p<0.001)*  The same trend was found in all other healthcare services studied; those who said they had less access were significantly likelier to have experienced discrimination. | |
| Rees et al. (25)  ***Wales*** | 2023 | | Analytical cross-sectional | | Can routine data be used to estimate the mental health service use of children and young people living on Gypsy and Traveller sites in Wales? A feasibility study | | 993 718 | | Data was used from the Adolescent Mental Health Data Platform. | | | | GTs could not be easily identified in routine health datasets through ethnicity coding.  GTs had significantly higher DNA rates at psychiatric follow-up appointments than non-Romas (36.9% vs. 15.1%)  No difference between GTs and non-GTs were seen in attendance rates at first psychiatric appointments.  Within GTs that are registered to a GP, access of health services for mental health is higher than non-GRTs. | |
|  |  |  |  |  |  |  | 802 Gypsy/Irish Traveller (GT)^b^ | |  |  |  |  |  |  |
| Silarova et al. (26)  ***Slovakia*** | 2014 | | Analytical cross-sectional | | Anxiety and sense of coherence in Roma and non-Roma coronary heart disease patients | | 607 | | **Anxiety:** Hospital Anxiety and Depression Scale (HADS) | | | | Coronary heart disease patients who were Roma experienced significantly more anxiety than those who were non-Roma (*p<0.01*), even after adjusting for age, sex, and socioeconomic status. | |
|  |  |  |  |  |  |  | 98 Roma | |  |  |  |  |  |  |
| Tanner et al. (27)  ***Ireland*** | 2022 | | Analytical cross-sectional | | Suicidal Ideation and Behaviors Among Irish Travellers Presenting for Emergency Care | | 1323 | | An anonymised database from a tertiary hospital was studied to find presentations to the emergency department of suicidal ideation (SI) or self-harm (SH). | | | | Travellers make up 4.8% of SI/SH presentations to the hospital despite making up just 1.6% of the population.  Travellers were more commonly diagnosed with depression and transferred for inpatient psychiatric treatment. | |
|  |  |  |  |  |  |  | 198 (all Irish Travellers)^b^ | |  |  |  |  |  |  |
| Tobin et al. (28)  ***Ireland*** | 2020 | | Qualitative | | Grief, Tragic Death, and Multiple Loss in the Lives of Irish Traveller Community Health Workers | | 11 (all Irish Travellers)^b^ | | Qualitative interviews and interpretative phenomenological analysis (IPA) were used. | | | | Normalization of suicide occurs in the Traveller community, due to a sense of fatalism about the act.  Fatalism results in constant worrying/anxiety over vulnerable ones’ well-being & anticipation of death. This fear is connected to shame/stigma, resulting in silence.  Lack of culturally appropriate bereavement counselling and inability to talk about stigmatized topics has an exacerbating effect on the Travellers’ grief and can lead to mental health issues. | |
| Tong et al. (29)  ***Ireland*** | 2021 | | Analytical cross-sectional | | Borderline personality disorder in Irish Travellers: a cross-sectional study of an ultra-high-risk group | | 51 (all Irish Travellers)^b^ | | Anonymised patient records from a community mental health team in Tuam, Galway, Ireland were used. | | | | Despite making up 8.4% of the Tuam population, 12.4% of all service users were Travellers.  77.8% of the 27 patients who were offered group psychotherapy declined this option. | |
| Toth et al. (30)  ***Hungary­­­­*** | 2018 | | Mixed-methods: analytical cross-sectional & qualitative | | Risk factors for multiple suicide attempts among Roma in Hungary | | | 150 | | **Depression:** Beck Depression Inventory (BDI)  **Hopelessness:** Beck Hopelessness Scale  Qualitative interviews were also used. | | | | Multiple attempters were found to have severe depression according to BDI scores, but only 51.6% of Roma multiple attempters had a diagnosed mood disorder compared to 72.7% of non-Roma multiple attempters (*p<0.001*).  Roma attempters were less likely to have suicidal intent (53.3% vs. 83.3%, *p<0.001*), meaning they were likelier to attempt suicide without the intent to die. 66.7% of Romas said they attempted to “escape from an unbearable situation”.  Less of the Roma attempters had planned their attempt in advance (6.7% vs. 68.5%, *p<0.001*), meaning they were likelier to attempt impulsively.  Romas reported 4.2x more suicide attempts in their life (3.5 vs. 0.8, *p<0.001*), meaning they were likelier to have multiple suicide attempts.  Smoking, suicide in the family, and being unemployed for >1 year were risk factors for suicidal behaviour in Romas, but not in non-Romas. |
|  |  |  |  |  |  |  |  | 90 Roma | |  |  |  |  |  |
| Vazsonyi et al. (31)  ***Czech Republic*** | | 2020 | | Analytical cross-sectional | | Neighborhood effects on internalizing and externalizing problems, and academic competence: a comparison of Roma and non-Roma adolescents | 369 | | | | **Depression:** depression subscale of the Weinberger Adjustment Inventory  **Anxiety:** anxiety subscale of the WAI  **Self-Esteem:** self-esteem subscale of the WAI | No significant differences were detected in depressive or anxious symptoms in Roma and non-Roma adolescents once neighbourhood effects were adjusted for.  Fear/concerns surrounding neighbourhood safety was a positive predictor for anxiety, depression, and low self-esteem. Furthermore, higher neighbourhood cohesion was a positive predictor for anxiety. | | |
|  |  |  |  |  |  |  | 239 Roma | | | |  |  |  |  |
| Villani et al. (32) | | 2021 | | Qualitative | | A qualitative study of the perceptions of mental health among the Traveller community in Ireland | 25 (all Irish Travellers)^b^ | | | | Qualitative interviews were used. | Positive mental health was associated with good social connections, and support in the community.  Poor mental health was associated with low socioeconomic status (employment, education, and housing disparities) and prejudice/discrimination. Many participants believed that poor mental health starts in childhood through discrimination at school.  Traveller men were more concerned with the impact of stigma/low income. Traveller women were more concerned with gender inequality.  The government’s policies over the last few decades are seen as criminalising the Traveller culture and way of life, leading to lack of self-worth.  Awareness/competence of Traveller culture was seen as necessary to provide meaningful mental healthcare to Traveller communities. | | |
| Villani et al. (33) | | 2024 | | Qualitative | | Implementing culturally appropriate recovery approaches in mental health services: Perspectives from the irish traveller community | 87 (all Irish Travellers)^b^ | | | | Qualitative interviews and participatory workshops were used. | Increasing Traveller cultural awareness and employing more Travellers in mental healthcare services (even as non-clinical roles) would help strengthen these services and build trust.  The government should involve Traveller groups/foundations when producing strategies aimed at tackling the issue of mental health in Traveller communities.  Non-Traveller mental health workers should receive training on Traveller culture, including interacting with people from this community through Traveller organisations. | | |

| Vorvolakos et al. (34)  ***Greece*** | 2010 | Analytical cross-sectional | Sociodemographic and clinical characteristics of Roma and non-Roma psychiatric outpatients in Greece | 254 | **Psychiatric disorder:** Structured Clinical Interview according to DSM-III-R  **Personality disorder:** International Personality Disorder Examination (IPDE)  **Psychopathological symptoms:** Derogatis Psychiatric Rating Scale and Global Pathology Index | Romas had significantly lower rates of psychotic disorders (30.3% vs. 14.3%, *p=0.004*) and bipolar disorders (10.6% vs. 2.5%, *p=0.022*), but significantly higher rates of depression (45.4% vs. 28.8%, *p=0.01*).  Romas had significantly higher scores on the DPRS for the domains of somatisation (3.35 vs. 1,.94), depression (3.73 vs. 2.81), anxiety (4.13 vs. 2.89), hostility (2.93 vs. 1.35), phobic anxiety (2.62 vs. 1.88), sleep disturbance (3.09 vs. 1.55), psychomotor retardation (3.09 vs. 1.93), hysterical behaviour (3.16 vs. 1.66), and abjection-disinterest (3.47 vs. 2.13). All *p<0.001*. |
| --- | --- | --- | --- | --- | --- | --- |
|  |  |  |  | 122 Roma |  |  |
| Watkinson et al. (35)  ***UK*** | 2021 | Analytical cross-sectional | Ethnic inequalities in health-related quality of life among older adults in England: secondary analysis of a national cross-sectional survey | 1 394 361 | **Anxiety/depression:** anxiety or depression subsection of Euroqol-5D (EQ5D)  **Health-related quality of life:** EQ5D | For both men and women, GTs scored the highest of all ethnicities on the EQ-5D subsection relating to anxiety or depression.  For both men & women, GTs had the worst EQ5D scores. The difference in EQ5D was equivalent to a 20-year increase in the age of the whole population. |
|  |  |  |  | 226 Gypsy/Irish Travellers (GT)^b^ |  |  |
| Zelko et al. (36)  ***Slovenia*** | 2015 | Analytical cross-sectional | Quality of Life and Patient Satisfaction with Family Practice Care in a Roma Population with Chronic Conditions in Northeast Slovenia | 574 (all Roma) | **Anxiety/depression:** self-reported presence of anxious/depressive symptoms  **Psychiatric diagnosis:** self-reported | 66.7% of female Romas had mental problems, compared with 33.3% of male Romas.  Issues with mobility, self-care, pain, & everyday activities were associated with a higher rate of mental health diagnoses. However, presence of anxiety & depression was the strongest indicator of a mental health diagnosis.  Anxious/depressive symptom & mental diagnoses groups had the worst health-related quality of life. |
| Zonda et al. (37)  ***Hungary*** | 1990 | Analytical cross-sectional | Suicide among Hungarian Gypsies | 790 | Interviews of suicide attempters were used. | Romas are less likely to complete suicide, but are more likely to attempt suicide, than non-Romas.  Less Roma suicide attempters had completed suicide after a follow-up time of 6-16 years than non-Roma attempters (1.0% vs. 3.3%).  64% of Romas, vs. 38% of non-Romas, said that the intent of their attempt was to frighten or appeal to others. |
|  |  |  |  | 105 Romas |  |  |

^a^ Mental Health Measurement refers to the measures used to determine mental health status.

^b^ Study is UK or Ireland based; as such, the relevant term (Gypsies and/or Travellers) has been used instead of Roma.

References

1. Abdalla S, Quirke B, Daly L, Fitzpatrick P, Kelleher C. All Ireland traveller health study: Differentials in cause-specific mortality between Irish travellers and the general population in the Republic of Ireland. Irish Journal of Medical Science. 2011;180(6 SUPPL. 1):S217.

2. Barrio-Forne N, Gasch-Gallen A. Companionship as a method to reduce anxiety in pregnant women hospitalized during their third trimester. Revista Da Escola de Enfermagem Da Usp. 2021;55:e03749.

3. Carrasco-Garrido P, Lopez de Andres A, Hernandez Barrera V, Jimenez-Trujillo I, Jimenez-Garcia R. Health status of Roma women in Spain. European Journal of Public Health. 2011;21(6):793-8.

4. Filakovska Bobakova D, Chovan S, Van Laer S. Perceived Stress of Mothers, Harsh Discipline, and Early Childhood Mental Health: Insights from a Cross-Sectional Study in Marginalized Roma Communities. International Journal of Public Health.69:1606721.

5. Gouva M, Mentis M, Kotrotsiou S, Paralikas T, Kotrotsiou E. Shame and Anxiety Feelings of a Roma Population in Greece. Journal of Immigrant & Minority Health. 2015;17(6):1765-70.

6. Goward PR, J.; Appleton, L.; Hagan, T. Crossing boundaries: identifying and meeting the mental health needs of Gypsies and Travellers. Journal of Mental Health. 2008;15(3):315-27.

7. Hayanga B, Stafford M, Saunders CL, Becares L. Ethnic inequalities in age-related patterns of multiple long-term conditions in England: Analysis of primary care and nationally representative survey data. Sociology of Health & Illness. 2023;25:25.

8. Heredia-Amador A, Calvo-Salguero A, Salinas JM, Gamella JF. Differences in the prevalence of depression in older Spanish Romany and non-Romany people and associated factors. Psychogeriatrics:The Official Journal of the Japanese Psychogeriatric Society. 2018;18(4):313-20.

9. Inel Manav A. An evaluation of early marriage and the mental state of Roma women: A cross-sectional study. Transcultural Psychiatry.61(1):107-17.

10. Kavalidou K, Daly C, McTernan N, Corcoran P. Presentations of self-harm and suicide-related ideation among the Irish traveller indigenous population to hospital emergency departments: Evidence from the National Clinical Programme for self-harm. Social Psychiatry and Psychiatric Epidemiology: The International Journal for Research in Social and Genetic Epidemiology and Mental Health Services. 2023;58(6):883-91.

11. Keogh B, Brady AM, Downes C, Doyle L, Higgins A, McCann T. Evaluation of a Traveller Mental Health Liaison Nurse: Service user perspectives. Issues in Mental Health Nursing. 2020;41(9):799-806.

12. Knipe D, Moran P, Howe LD, Karlsen S, Kapur N, Revie L, et al. Ethnicity and suicide in England and Wales: a national linked cohort study. Lancet Psychiatry. 2024;11(8):611-9.

13. Kohler IV, Preston SH. Ethnic and Religious Differentials in Bulgarian Mortality, 1993-98. Population Studies. 2011;65(1):91-113.

14. Kolarcik P, Geckova AM, Orosova O, van Dijk JP, Reijneveld SA. To what extent does socioeconomic status explain differences in health between Roma and non-Roma adolescents in Slovakia? Social Science & Medicine. 2009;68(7):1279-84.

15. Kozubik M, van Dijk JP, Rac I. Health Risks Related to Domestic Violence against Roma Women. International Journal of Environmental Research & Public Health [Electronic Resource]. 2020;17(19):24.

16. Lee EJ, Keyes K, Bitfoi A, Mihova Z, Pez O, Yoon E, et al. Mental health disparities between Roma and non-Roma children in Romania and Bulgaria. BMC Psychiatry. 2014;14:297.

17. Linehan S, Duffy D, O'Neill H, O'Neill C, Kennedy HG. Irish travellers and forensic mental health. Irish Journal of Psychological Medicine. 2002;19(3):76-9.

18. Lopez JA, Garcia RF, Marti TS. Drugs and Mental Health Problems among the Roma: Protective Factors Promoted by the Iglesia Evangelica Filadelfia. International Journal of Environmental Research & Public Health [Electronic Resource]. 2018;15(2):14.

19. Malone KM, McGuinness SG, Cleary E, Jefferies J, Owens C, Kelleher CC. Lived Lives: A Pavee Perspective. An arts-science community intervention around suicide in an indigenous ethnic minority. Wellcome Open Research. 2017;2:27.

20. McGorrian C, Frazer K, Kelleher CC. Do Irish travellers pose a paradox in terms of healthcare access and engagement? findings from the all Ireland traveller health study (AITHS). Irish Journal of Medical Science. 2011;180(6 SUPPL. 1):S217.

21. Olah B, Biro E, Kosa K. Residence in segregated settlements (colonies) rather than Roma identity increases the risk of unfavourable mental health in Hungarian adults. Frontiers in Public Health.11:1205504.

22. O'Sullivan K, Brady AM, Downes C, Higgins A, Doyle L, McCann T, et al. The role and activities of the Traveller Mental Health Liaison Nurse: Findings from a multi-stakeholder evaluation. International Journal of Mental Health Nursing. 2021;30(6):1664-73.

23. Parry G, Van Cleemput P, Peters J, Walters S, Thomas K, Cooper C. Health status of Gypsies and Travellers in England. Journal of Epidemiology & Community Health. 2007;61(3):198-204.

24. Quirke B, Heinen M, Fitzpatrick P, McKey S, Malone KM, Kelleher C. Experience of discrimination and engagement with mental health and other services by Travellers in Ireland: Findings from the All Ireland Traveller Health Study (AITHS). Irish Journal of Psychological Medicine. 2022;39(2):185-95.

25. Rees S, Fry R, Davies J, John A, Condon L. Can routine data be used to estimate the mental health service use of children and young people living on Gypsy and Traveller sites in Wales? A feasibility study. PLoS ONE [Electronic Resource]. 2023;18(2):e0281504.

26. Silarova B, Nagyova I, Van Dijk JP, Rosenberger J, Reijneveld SA. Anxiety and sense of coherence in Roma and non-Roma coronary heart disease patients. Ethnicity & Health. 2014;19(5):500-11.

27. Tanner B, Doherty A. Suicidal Ideation and Behaviors Among Irish Travellers Presenting for Emergency Care: Ethnicity as a Risk Factor. Crisis. 2022;43(2):149-56.

28. Tobin M, Lambert S, McCarthy J. Grief, Tragic Death, and Multiple Loss in the Lives of Irish Traveller Community Health Workers. Omega - Journal of Death & Dying.81(1):130-54.

29. Tong K, Costello S, McCabe E, Doherty AM. Borderline personality disorder in Irish Travellers: a cross-sectional study of an ultra-high-risk group. Irish Journal of Medical Science. 2021;190(2):735

EP - 40.

30. Toth MD, Adam S, Zonda T, Birkas E, Purebl G. Risk factors for multiple suicide attempts among Roma in Hungary. Transcultural Psychiatry. 2018;55(1):55-72.

31. Vazsonyi AT, Liu D, Beier J, Blatny M. Neighborhood effects on internalizing and externalizing problems, and academic competence: a comparison of Roma and non-Roma adolescents. International Journal of Public Health.65(8):1383-92.

32. Villani J, Barry MM. A qualitative study of the perceptions of mental health among the Traveller community in Ireland. Health Promotion International. 2021;36(5):1450-62.

33. Villani J, Kuosmanen T, McDonagh M, Barry MM. Implementing culturally appropriate recovery approaches in mental health services: Perspectives from the irish traveller community. Irish Journal of Psychological Medicine. 2024:No-Specified.

34. Vorvolakos T, Samakouri M, Tripsianis G, Tsatalmpasidou E, Arvaniti A, Terzoudi A, et al. Sociodemographic and clinical characteristics of Roma and non-Roma psychiatric outpatients in Greece. Ethnicity & Health. 2012;17(1-2):161-9.

35. Watkinson RE, Sutton M, Turner AJ. Ethnic inequalities in health-related quality of life among older adults in England: secondary analysis of a national cross-sectional survey. The Lancet Public Health. 2021;6(3):e145-e54.

36. Zelko E, Svab I, Rotar Pavlic D. Quality of Life and Patient Satisfaction with Family Practice Care in a Roma Population with Chronic Conditions in Northeast Slovenia. Zdravstveno Varstvo. 2015;54(1):18-26.

37. Zonda T, Lester D. Suicide among Hungarian Gypsies. Acta Psychiatrica Scandinavica. 1990;82(5):381-2.
